# Benzodiazepine Dependence: Clinical and Molecular Aspects, Preventive Strategies and Therapeutic Approaches

**DOI:** 10.3390/ijms27031430

**Published:** 2026-01-31

**Authors:** Francisco Navarrete, Marta Marín-Mayor, Lorena Martínez-Hostyn, Gabriel Rubio, Jorge Manzanares

**Affiliations:** 1 Instituto de Neurociencias, Universidad Miguel Hernández-CSIC, Avda. de Ramón y Cajal s/n, San Juan de Alicante, 03550 Alicante, Spain; lorena.martinezh@umh.es; 2 Red de Investigación en Atención Primaria de Adicciones, Instituto de Salud Carlos III, MICINN and FEDER, 28029 Madrid, Spain; marta.marin@salud.madrid.org (M.M.-M.); grubiovalladolid@gmail.com (G.R.); 3 Instituto de Investigación Sanitaria y Biomédica de Alicante (ISABIAL), 03010 Alicante, Spain; 4 Instituto de Investigación (i+12), Hospital Universitario 12 de Octubre, 28041 Madrid, Spain; 5 Department of Psychiatry, Complutense University of Madrid, 28040 Madrid, Spain

**Keywords:** benzodiazepine, pharmacology, clinical indications, tolerance, dependence, withdrawal, molecular neurobiology, prevention, therapeutic strategies

## Abstract

Benzodiazepines (BZDs) are globally prevalent psychotropic substances valued for their anxiolytic, hypnotic, anticonvulsant, and myorelaxant properties. Pharmacologically, they act as positive allosteric modulators of the ionotropic GABA_A_ receptor, enhancing inhibitory synaptic transmission. However, prolonged use poses a significant public health concern, risking adverse effects such as cognitive impairment, motor incoordination, tolerance, and physical dependence. The development of tolerance is mediated by complex neurobiological changes, notably the downregulation of GABA_A_ receptor subunits and a compensatory sensitization of excitatory glutamatergic systems. Effective management of established dependence requires comprehensive psychological intervention coupled with pharmacological substitution (switching to a long-acting BZD) and gradual dose tapering. Preventive measures are complex, emphasizing short-term prescriptions, minimum effective dosing, and selecting non-pharmacological or alternative pharmacological agents, such as SSRIs/SNRIs, to mitigate the risk of developing tolerance and dependence. This expert review aims to compile the most relevant, representative, and recent literature summarizing the pharmacology, clinical indications, adverse effects, misuse, and abuse of BZDs that ultimately lead to BZD use disorder (BUD). It also details the involved neurobiological mechanisms and discusses critical preventive and therapeutic strategies, providing readers with the main aspects to consider for addressing this global public health problem.

## 1. Introduction

Worldwide, the use of benzodiazepines (BZDs) has recently increased, with Spain at the top of the list [1]. According to the latest report of the International Narcotics Control Board (United Nations, 2023), BZDs account for the majority of the production, trafficking and consumption of psychotropic substances under international control [2]. A descriptive cross-sectional study showed that increased use of BZDs and Z-drugs was statistically associated with higher Gross Domestic Product and increased prevalence of anxiety, neurological disorders (including Alzheimer’s disease, Parkinson’s disease, and headache), chronic respiratory and cardiovascular diseases, diabetes, and cancer [3].

Notably, the significant increase in the prevalence of anxiety disorders may explain much of the elevated use of BZDs. Indeed, 45.82 million incidents and 301.39 million prevalent cases of anxiety disorders were estimated worldwide in 2019, resulting in 28.68 million disability-adjusted life years (DALYs). Although the overall age-standardized burden rate of anxiety disorders has remained stable over the past three decades, the most recent absolute number has increased by 50% according to the latest Global Burden of Disease results [4]. The COVID-19 pandemic has played a significant role among the various triggers. In this sense, a cohort study showed an increase in BZD prescriptions from January 2020 to April 2020 during the first wave of COVID-19, suggesting that mitigation measures, including social isolation and stay-at-home orders, may have had a negative impact on mental health, particularly among women [5]. Indeed, the prevalence rate of all forms of depression, anxiety, stress, sleep problems, and psychological distress in the general population was found to be higher during and after the COVID-19 pandemic [6].

BZDs have significant clinical utility due to their anxiolytic, hypnotic, anticonvulsant and myorelaxant properties. However, there is a major public problem with the inappropriate use of these drugs, as they are often misprescribed or not monitored [7,8,9]. They should not be administered for more than 8–12 weeks, and the dose should be gradually increased and decreased, conditions that are often not met. Prolonged use of BZDs can lead to a wide range of adverse effects, including excessive sedation, cognitive impairment, or motor incoordination, as well as the development of tolerance and, eventually, dependence. Prolonged misuse of BZDs increases the risk of developing a BZD use disorder (BUD), which is classified as a ‘sedative, hypnotic or anxiolytic use disorder’ (SHD) according to the diagnostic classification in the latest version of the Diagnostic and Statistical Manual of Mental Disorders (DSM-5-TR) [10].

The therapeutic approach to patients who have developed criteria for BUD is very complex, heterogeneous, and limited by the lack of drugs with specific indications. It is essential to guide and motivate the patient appropriately and to choose the type of pharmacological approach according to possible comorbidities (psychiatric or addictive) and, particularly, the symptomatology that appears after the cessation or withdrawal of consumption, which can be life-threatening without appropriate treatment. In this regard, the first step in treating BZD withdrawal involves a combination of psychological interventions and pharmacological substitution, with gradual dose reduction and selection of BZDs with a long half-life and anticonvulsant properties [11].

This expert review aims to summarize the pharmacology, clinical indications, adverse effects, misuse, and abuse of BZDs that ultimately lead to BUD, as well as the neurobiological mechanisms involved in the negative consequences of long-term BZD use. It also discusses both preventive and therapeutic strategies to address this global public health problem.

## 2. A Brief Review of BZDs’ Pharmacology

BZDs are currently the most widely used anxiolytic and hypnotic drugs and have replaced their predecessors, the barbiturates, because they are safer and more effective. They act selectively on ionotropic γ-aminobutyric acid type A receptors (GABA_A_), which are primarily responsible for inhibitory synaptic transmission throughout the central nervous system (CNS). They bind specifically to a regulatory site on the receptor, distinct from the GABA binding site, and act as positive allosteric modulators, increasing the likelihood that the Cl^−^ channel opens in response to GABA. Not only classical BZDs bind to this receptor, but also non-BZD molecules such as cyclopyrrolones (zopiclone, eszopiclone), imidazopyridines (zolpidem), pyrazolopyridines (zaleplon), or β-carbolines (abecarnil) [12].

**Figure 1:**
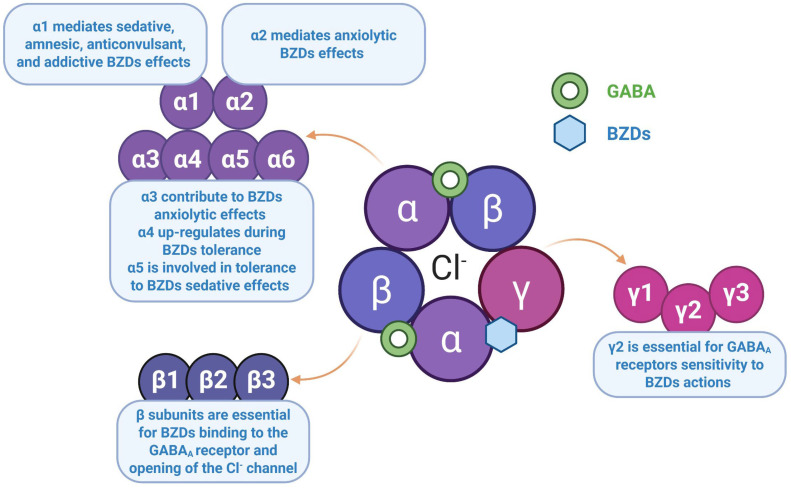
Representation of a GABA_A_ receptor, a transmembrane protein composed of five subunits that are associated to form a Cl^−^ permeable ion channel at its center. Generally consisting of two α, two β and one γ subunit, BZDs bind to a modulating site of the GABA_A_ receptor between the α and γ subunits, different from GABA binding in the β subunit. The sensitivity, binding and clinical ac-tions of BZDs on GABA_A_ receptors depend on the presence of specific α, β and γ subunits as detailed in the figure.

The GABA_A_ receptor is a member of the ion channel receptor family. It is a transmembrane protein composed of five subunits that are associated with the formation of a Cl^−^ permeable ion channel at its center (Figure 1). To date, seven subunit types have been cloned, each with several subtypes (α 1–6, β 1–3, γ 1–3, δ, ɛ, θ, ρ 1–3), giving the GABA_A_ receptor an extraordinary structural diversity that offers the possibility of designing molecules with different affinities for the various receptor subtypes, which would confer a specific pharmacological profile. In vivo, most receptors are composed of a combination of two α, two β and one γ subunit (Table 1). GABA binding occurs at the β subunit, whereas BZDs bind at the interface between the α and γ subunits. Receptors containing the α1, α2, α3 or α5 subunits in combination with any of the β and γ2 subunits are characterized by sensitivity to modulation by BZDs. It is now accepted that the pharmacological peculiarity of BZDs lies in the structural diversity of GABA_A_ receptors, as well as in their differential brain regional expression [13,14,15].

Studies in genetically modified mice have been instrumental in identifying the correlation between the GABA_A_ α receptor subtype and its involvement in the primary effects of BZDs. Thus, receptors containing the α1 subunit mediate sedative, amnesic, anticonvulsant, addictive and alcohol-potentiating effects, but are not involved in myorelaxant or anxiolytic effects. In contrast, receptors containing the α2 subunit mediate anxiolytic effects, although α3 may also contribute. The α5 subunits, located in the hippocampus, modulate learning and memory; they have also been implicated in the development of tolerance to the sedative effects of BZDs. It has been suggested that the loss of consciousness and immobility associated with general anesthesia are mediated by the β2 and β3 subunits, respectively. Numerous BZD receptor ligands with intrinsic activity at α2 and/or α3 receptors, but without α1 activity, have been developed and exhibit anxiolytic effects without sedation. However, none of these molecules have progressed beyond phase III clinical trials due to adverse effects [13,17,18].

The behavioral and sedative effects of BZDs can be attributed in part to the potentiation of GABAergic pathways that regulate the firing of monoamine-containing neurons, which are known to promote behavioral arousal and are essential mediators of the inhibitory effects of fear and punishment on behavior. Inhibitory effects on muscular hypertonia or the spread of seizure activity can be attributed to potentiation of inhibitory GABAergic circuits at various brain levels [19].

The fundamental differences between the various BZDs are pharmacokinetic, with onset of action and duration of clinical effect being the most critical parameters to consider in this group of drugs. They bind strongly to plasma proteins, and their high lipid solubility allows many to accumulate gradually in body fat or readily cross the blood–brain barrier. Although the most common route of administration is oral, they can be given intravenously in certain situations (diazepam for status epilepticus or midazolam for anesthesia). Rectal administration may also be helpful. Intramuscularly, most BZDs are absorbed erratically and slowly, especially chlordiazepoxide and diazepam, probably because they are concentrated in adipose tissue.

On the other hand, the majority of BZDs are metabolized in the liver by cytochrome P-450 isoenzymes and are ultimately excreted in the urine as glucuronide conjugates. The duration of action of different BZDs varies widely, and the half-life is used as a way of classifying them. Short-acting compounds are used as hypnotics, and long-acting compounds are more useful as anxiolytics and anticonvulsants. Accordingly, a classical classification of BZDs into short-, intermediate- and long-acting has been established based on the half-life (t½) of elimination value and that of the active metabolites (Table 2). However, this should not be the only criterion considered, as the duration of a given effect depends on the time during which the drug concentration remains above a threshold, which is strongly influenced by redistribution [12,20].From a clinical pharmacology perspective, non-pharmaceutical or ‘designer’ BZDs (DBZDs) are a concerning subset of new psychoactive substances (NPS) as they are synthetic analogues of licensed BZDs whose pharmacokinetic and pharmacodynamic profiles are largely unknown. Since the mid-2000s, there has been an increase in the number and availability of DBZDs on the European drug market and, increasingly, elsewhere. Thirty new BZDs have appeared on the drug market. More than 80% of these were first detected between 2014 and 2020. Despite this relatively large number, the European market is dominated by a few substances. Over the past few years, etizolam and flualprazolam in particular have become increasingly important in the new BZD market in certain European countries, particularly in the production of counterfeit diazepam and alprazolam tablets [21]. These new psychoactive substances contain a BZD core and include closely related compounds (e.g., thienodiazepines). They are not controlled under the international drug control system. They should therefore be approached with caution due to the dangers associated with their uncontrolled use, given that their pharmacodynamic and pharmacokinetic profiles are similar to those of clinically used BZDs. DBZDs act as positive allosteric modulators at the GABAA receptor, producing sedation, anxiolysis, hypnosis, muscle relaxation, amnesia and euphoria. However, subtle structural modifications can markedly alter their receptor affinity, potency, onset and duration of action, often making them substantially more potent and unpredictably longer-acting than their pharmaceutical counterparts [22]. As these compounds are manufactured illicitly without pharmaceutical quality control and are often marketed online or sold as counterfeit prescription BZDs, users are frequently unaware of the substance or dose they are consuming. This undermines any reliable dose–response expectations and substantially increases the risk of unintentional overdose and toxicity [23]. Clinically, the management of DBZD intoxication is complicated by the fact that it is not detected on standard toxicology screens and by the unpredictable response to antagonists such as flumazenil, particularly in chronic users, where precipitated withdrawal can be dangerous [24]. The absence of standardized dosing, the potential for active metabolites with long elimination half-lives and frequent combination with other central nervous system depressants, such as opioids or alcohol, amplifies the risk of profound sedation, respiratory depression and fatal adverse events [25]. Of special importance in this context is the use of the so-called ‘benzo-dope’, which consists of a synthetic opioid (mainly illicit fentanyl) mixed with a DBZD [26]. All these factors, therefore, make DBZDs a significant public health concern, exemplifying the challenges posed by NPS in emergency and addiction medicine [27].

Adverse reactions are often due to dosage irregularities. The most common are unwanted increases in pharmacological effects, including sedation, confusion, disorientation, dysarthria, ataxia, drowsiness, reduced motor coordination, and an inability to react quickly to stimuli requiring a motor or verbal response. These disturbances can be hazardous when driving or operating machinery. Accumulation of long half-life BZDs or BZDs with active metabolites, especially with prolonged treatment, may cause these types of adverse reactions, which are less usual with short half-life BZDs. Significantly, as CNS depressants, their combination with other substances with the same effect, such as alcohol, barbiturates, antihistamines, sedatives or opioids, may reduce the safety margin of these drugs and can be life-threatening. In general, adverse reactions involving the CNS are more common in aged patients and those with brain disorders due to increased sensitivity or reduced elimination. In this type of patient, the anterograde amnesia that BZDs can induce, which may lead to repeated doses due to forgetfulness, must be considered. Rapid intravenous administration may cause hypotension, respiratory depression and even cardiac arrest [13,20].

Overdosage may result in the adverse effects described above, including hazardous respiratory depression, which may lead to coma. In the event of acute intoxication, administration of the antagonist flumazenil at a dose of 0.2–4 mg is indicated. Continued use of BZDs leads to tolerance to the sedative and anticonvulsant effects due to accumulation. Tolerance is crossed with alcohol and other sedative drugs. They can also cause psychological and physical dependence, even at low doses, with a withdrawal syndrome that develops slowly after drug cessation and is characterized by the onset of anxiety, insomnia, restlessness, irritability, tremors, headache, dizziness, confusion, sensory disturbances and somatic symptoms such as palpitations, hyperventilation, or irritable bowel. These symptoms are often difficult to distinguish from a relapse of the original anxiety state. The primary syndrome consists of delirium, hallucinations, depersonalization, and convulsions. The higher the dose and the longer the treatment duration, the more severe the symptoms. About 35% of patients treated with BZDs for more than 4 weeks develop physical dependence. The BZDs with the most significant potential for dependence (in terms of severity and latency of withdrawal) are those with the highest potency and lowest elimination t1/2. Prescribing low doses and administering them intermittently greatly minimizes the problems of tolerance and dependence. In any case, it is recommended not to prolong treatment beyond 8–12 weeks. Clinical studies suggest that prolonged use of BZDs over many years not only fails to control anxiety but may worsen it [13,20]. All relevant safety aspects are discussed in more detail in the following sections of this review.

## 3. Clinical Indications of BZDs

BZDs are one of the most widely prescribed drugs worldwide. They have a rapid onset of action, significant efficacy, good tolerability and relative safety in short-term applications. As mentioned in the Section 2, BZDs bind to the central GABA_A_ receptor at both postsynaptic and presynaptic neurons. They act as positive allosteric modulators, enhancing GABA’s action at the GABA_A_ receptor, allowing it to exert a more potent effect.

### 3.1. Anxiety Disorders and Anxiety Symptoms

BZDs have a rapid onset of anxiolytic effect. Therefore, BZDs have traditionally been used in the treatment of anxiety disorders and anxiety symptoms related to other mental disorders. Several meta-analyses have found that BZDs effectively treat anxiety disorders [29,30,31]. The two anxiety disorders in which the efficacy and safety of BZDs have been explored more frequently are generalized anxiety disorder (GAD) and panic disorder (PD). Several meta-analyses have found that BZDs are more effective than placebo [32,33], psychological interventions [33] and antidepressants, specifically selective serotonin reuptake inhibitors (SSRIs) [34], in treating GAD symptoms.

On the other hand, PD is a common and acute mental disorder on the anxiety spectrum, characterized by recurrent and unpredictable panic attacks consisting of a wave of intense fear or anxiety that reaches a peak within a few minutes. These behaviors are often accompanied by several physical symptoms, including palpitations, chest pain, breathlessness, sweating, trembling, dizziness, flushing, stomach churning, and faintness. In the most recent Cochrane Review exploring the effectiveness of BZDs, SSRIs, selective noradrenaline reuptake inhibitors (SNRIs), tricyclic antidepressants (TCAs) and mono-amine oxidase inhibitors (MAOIs) in the treatment of PD in adults, different BZDs (diazepam, alprazolam, and clonazepam) were found to be the most effective treatments compared to other pharmacological agents. Specific BZDs also demonstrated efficacy in terms of remission (alprazolam) and reductions in panic scale scores (clonazepam), as well as decreases in the frequency of panic attacks (clonazepam and alprazolam) and agoraphobia (diazepam) [35].

Despite the demonstrated efficacy of BZDs in the treatment of anxiety disorders, given their side-effect profile and their potential risk for the development of tolerance and dependence, there is a consensus in recommending BZDs to be used as an adjunctive treatment along with other first-line medications for anxiety disorders, such as SSRIs or SNRIs. BZDs are recommended only in the short-term (no longer than 8–12 weeks) while they exert their onset of action, for acute episodic anxiety or as an abortive treatment for panic attacks [31,36].

### 3.2. Sleep Disorders (Insomnia)

Insomnia is a common disorder characterized by sleep disturbances, including difficulty falling asleep, maintaining sleep, or premature awakening, or a combination of these. These disturbances are associated with daytime sleepiness, increased fatigue, reduced alertness, mood disruptions, diminished cognitive abilities, including attention, focus, and memory issues, as well as impaired functioning [37,38].

BZDs affect sleep architecture, increasing the N2 phase of non-rapid eye movement (NREM) sleep and decreasing the N3 and N4 phases of NREM sleep, resulting in a reduction in time spent in rapid eye movement (REM) sleep during nocturnal sleep. It has been proposed that the effects of BZDs on the N2 phase of NREM sleep may lead to a subjective improvement of sleep quality with no awakenings [39].

Although cognitive behavioral therapy and sleeping hygiene measures are considered first-line treatments for chronic insomnia, BZDs, among other pharmacological treatments, have also been recommended for treating insomnia. Meta-analyses exploring the efficacy and tolerability of pharmacological treatments for adult insomnia have found that BZDs are more effective than placebo in treating acute insomnia [40], particularly in improving subjective sleep quality [37]. However, there is no data regarding their usefulness in long-term insomnia, and their tolerability and safety profile are unfavorable [37,40].

Different BZDs can be chosen depending on the type of insomnia. High lipophilic BZDs, with a faster onset of action, as well as short-half-life BZDs, will be preferable for initial insomnia.

### 3.3. Agitation

Agitation is characterized by excessive motor activity, usually non-productive and repetitious, and is associated with a feeling of inner tension that can potentially escalate into aggressive and violent behaviors. It is associated with many psychiatric illnesses (schizophrenia, bipolar disorder and personality disorder), substance use disorders (SUD) (intoxication or abstinence) and medical conditions (neurological or metabolic diseases) [41].

BZDs are commonly used in the pharmacological treatment of acute agitation. They can be administered orally or intramuscularly, in monotherapy or combined with first- or second-generation antipsychotics [41,42]. Specific BZDs studied in acute agitation include lorazepam, clonazepam, flunitrazepam and midazolam. Generally, midazolam appears to have a more rapid onset of action.

### 3.4. Catatonia

Catatonia is a neuropsychiatric syndrome characterized by abnormal movements, behaviors, and withdrawal, caused by several medical (infections, endocrine, metabolic and neurologic) and psychiatric (mood and psychotic) disorders [43].

BZDs are the first-line treatment for catatonia, with lorazepam being the most widely studied medication at doses ranging from 2 to 16 mg/day [44]. Other BZDs, such as diazepam, midazolam, alprazolam, oxazepam, flunitrazepam, temazepam and clonazepam, have been effective in the treatment of catatonia [43]. Oral administration is desirable if possible. However, if the patient’s mental health state is too compromised, parental or intravenous methods can also be used. BZDs are effective in treating catatonia, with response and remission rates ranging from 60% to 100%.

### 3.5. Alcohol Withdrawal Syndrome

Alcohol withdrawal syndrome (AWS) occurs in chronic alcohol users who develop physical alcohol dependence after a rapid reduction or cessation in alcohol consumption, and it is due to an imbalance between glutamate (increased) and GABA (reduced) activity in the CNS. It begins within 6–24 h after the last alcohol consumption.

BZDs are considered the first-line treatment for AWS, as they are the most extensively studied drugs for this condition. They have demonstrated efficacy over placebo and other alternative treatments for treating seizures and *delirium tremens*, with no significant adverse events or dropouts [45,46].

Despite BZDs having proven their usefulness in the management of AWS, several potential complications must be considered: abuse liability; cognition impairment; respiratory depression; psychomotor retardation; BZD-delirium; significant interactions with alcohol, opioids and other CNS depressant drugs; and craving increase causing early relapse to alcohol use [47,48].

### 3.6. Other Psychiatric Disorders

BZDs can also be used in other psychiatric disorders. In depressive disorders, BZDs have been considered a valuable treatment for depression, mainly when it is associated with anxiety [49]. In patients with anxious depression, adding a BZD to an antidepressant can produce relief from both anxiety and irritability/dysphoria and can block the initial anxious activation associated with the early stages of treatment with antidepressants [50]. A Cochrane review evaluated the use of antidepressants plus a BZD versus antidepressants in monotherapy in the treatment of major depressive disorder (MDD) and found that during the first 4 weeks of treatment, the combination treatment was more effective in reducing depressive and anxiety symptoms, associated with higher response and remission rates and fewer dropouts because of adverse events [51].

### 3.7. Other Medical Conditions

In addition to psychiatric indications, BZDs can be used in other medical conditions. BZDs are considered first-line treatments in urgent seizure emergencies, including status epilepticus, acute seizures, cluster seizures and seizures due to alcohol and BZD withdrawal [36]. In addition, some BZDs, such as midazolam and fentanyl, administered intravenously, are indicated for preoperative sedation, induction of general anesthesia and sedation of ventilated individuals in intensive care units.

## 4. Complications Associated with BZDs Use

Chronic use of BZDs can lead to several medium- and long-term complications, especially in the older population. Within these complications, with special emphasis on the elderly, the most relevant are:

### 4.1. Psychomotor and Cognitive Effects

Psychomotor and cognitive side effects have been reported associated with the use of BZDs. Specific cognitive areas affected by the use of BZDs include psychomotor performance and speed, learning, acquisition of new knowledge (anterograde memory), visuospatial perception, attention, and immediate memory [50,52].

### 4.2. Cognitive Decline and Dementia

Another issue of special concern is the association of BZD use and the future development of cognitive decline and/or dementia. Data on whether BZDs increase the risk of cognitive impairment or dementia is controversial [50,53]. Several case–control [54,55,56,57,58,59], cohort [60,61] and longitudinal studies [62,63,64,65] have concluded that BZD consumption constitutes a risk factor for dementia, Alzheimer’s disease and cognitive impairment in aged patients. This risk is increased when using two or more BZDs [64], at higher doses, and for a longer time but may be reversible upon discontinuation of the BZD [55].

Most of the meta-analyses that have explored the relationship between long-term BZD use and the risk of dementia conclude that long-term BZD users have an increased risk of dementia compared with BZD non-users [66,67,68,69,70]. It has been reported that chronic BZD users have a 1.23- to 2-fold increased risk of developing dementia compared with BZD non-users [67,68,69,70,71]. This has been described as “ever” for recent or past BZD users [66]. Many of these meta-analyses have found that this risk increases with extended treatment periods [69,70], higher BZD doses [66], and long-acting BZDs [69,70]. Also, diabetes, hypertension, cardiac disease and taking statin drugs have been associated with increased risk of dementia in these patients [67]. However, most of these meta-analyses also note that their results should be interpreted with caution due to significant statistical and clinical heterogeneity among the selected studies, as well as potential bias arising from the lack of adjustment for confounding factors [67,68].

Several hypotheses have tried to explain how BZDs can lead to dementia. Firstly, it has been proposed that, due to their mechanism of action (positive allosteric modulators of the GABA_A_ receptor), they could induce synaptic inhibition in the CNS and may reduce the ß-secretase and γ-secretase, which are protective against cognitive impairment.

However, other studies fail to find a significant association between BZD use and the development of dementia or Alzheimer’s disease [72,73,74,75,76,77]. Some studies have suggested that chronic BZD use may be associated with poorer cognitive performance but not accelerated cognitive decline with age [78].

In addition, neuroimaging studies in non-demented older adults using amyloid Positron Emission Tomography (PET) have found that BZD users present with reduced cortical ß-amyloid expression, especially with short-acting BZDs, with no differences in global cognitive function [79,80]. The most recent meta-analysis published to date did not find a significant relationship between chronic BZD use and the development of dementia or Alzheimer’s disease [81].

In conclusion, and based on the current evidence, prolonged exposure to BZDs may be an indicator of an increased risk of dementia rather than a direct cause of its development, including the associated neurodegenerative processes.

### 4.3. Risk of Falls

Several meta-analyses have found that the use of BZDs is associated with an increased risk of falls, particularly in older adults [50,82,83]. Some BZD side effects, such as confusion, dyskinesia and delayed reaction time, can lead to this increase [83].

### 4.4. Impaired Driving

Because of increased sedation and impaired psychomotor skills, BZDs impair complex skills such as driving or operating machinery [50,52]. A meta-analysis comprising 13 case–control, 8 cohort, and 69 experimental studies revealed that BZDs were associated with a 60% to 80% increased risk of traffic accidents and a 40% increase in accident liability. In addition, combining the use of BZDs and alcohol was associated with a 7.7-fold increase in accident risk. Drivers < 65 years of age showed a greater BZD-associated accident risk.

### 4.5. Disinhibition

Paradoxical behaviors have been associated with BZD use. This disinhibitory effect is characterized by increased anxiety, acute excitement, hyperactivity, agitation, impulsivity, hostility, and aggressive behavior [50,52].

### 4.6. Pregnancy and Breastfeeding

Several meta-analyses have investigated the relationship between BZD exposure during pregnancy and teratogenicity. Most of them found that prenatal BZD exposure was significantly associated with an increased risk of spontaneous abortion, preterm birth, low birth weight, small for gestational age, low Apgar score, Neonatal Intensive Care Unit admission and induced abortion [84,85,86].

On the one hand, some meta-analyses have failed to find a significantly increased risk of congenital malformations or cardiac malformations following prenatal exposure to BZDs [85,87]. In contrast, others conclude that exposure to BZDs during the first trimester of pregnancy is associated with an increased risk of congenital malformations and cardiac malformations [86]. The concurrent use of BZDs and antidepressants during pregnancy is associated with a significantly increased risk of congenital malformations [87]. It has also been described as a syndrome known as “floppy infant syndrome” characterized by muscle laxity, failure to suckle and oversedation. In addition, approximately 2 weeks after birth, the infant can experience a withdrawal syndrome consisting of continued difficulty feeding, high-pitched cries, hyperexcitability and developmental retardation [36], which could have an impact on later stages of life.

### 4.7. Risk of Death

BZDs are safe drugs with a high therapeutic index, rarely lethal unless they are combined with other depressant CNS drugs such as alcohol or opioids [36,50,88]. A recent study concluded that during January–June 2020, more than 90% of deaths associated with BZDs also involved opioids, mainly illicit fentanyl (more than 60%) [89]. In addition, a slight increase in the risk of harm among patients with stable long-term prescription BZD treatment who appear to discontinue relative to continuing treatment, including those with and without recent opioid prescriptions [90].

The risk of suicide was analyzed in a study with 4,762,438 users of opioids, BZDs, and both drugs concomitantly. The results revealed that neither drug showed a small association for opioids but a more significant association for BZDs with suicide attempt, intentional self-harm, and drug overdose [91]. Furthermore, a recent systematic review revealed that concurrent use of BZDs and opioids significantly elevates the risk of suicide attempts and intentional self-harm, and BZD use was linked to increased suicide risk in vulnerable groups with pre-existing mental health conditions [92].

## 5. Misuse and Abuse of BZDs—Clinical Aspects

### 5.1. BZD Intoxication

BZDs are associated with different intensity intoxication symptoms. According to the DSM-5-TR [10], BZD intoxication symptoms include slurred speech, incoordination, unsteady gait, nystagmus, impaired cognition (memory and attention), and stupor or coma. Mild intoxication symptoms include apathy, dry mouth, hypotonia, ataxia, poor motor coordination, dysarthria, nystagmus, disorientation, dizziness, vertigo, blurry vision, and somnolence.

### 5.2. Tolerance to BZDs’ Effects

Tolerance refers to a progressive reduction in the BZD’s effect or an increase in the dosage needed to produce the same effect. BZD tolerance develops as a result of long-term modifications to the intraneural gene expression and function of GABA receptors, including the downregulation of BZD binding sites on GABA_A_ receptors, uncoupling of the allosteric linkage of the BZD-GABA_A_ receptor complex, and changes in receptor subunit turnover. Furthermore, chronic BZD-induced enhancement of GABA neurotransmission results in compensatory sensitization of excitatory neurotransmitter systems, such as glutamate [93].

The tolerance phenomenon associated with the continued use of benzodiazepines affects not only the sedative, hypnotic and anticonvulsant effects, but also myorelaxation. Conversely, tolerance to the anxiolytic effects is delayed and inconsistent in animal studies and appears to be modest or even absent in humans [93,94]. Similarly, the amnestic effects associated with chronic BZD treatment are not reduced, which is particularly dangerous in the case of elderly patients [81].

The development of tolerance to BZDs could lead to misuse and abuse, with an escalation of dose over the previous one to achieve the same pharmacological effects. However, escalating BZDs has been associated with their long-term use, which is uncommon [94]. It is important to note that developing tolerance to BZDs does not necessarily lead to physical dependence on them. In this way, BZD tolerance and withdrawal seem to be two independent phenomena [94].

### 5.3. Discontinuation Syndromes

After the cessation of BZD consumption, three different types of syndromes can develop [50,52,94] (Table 3):

- **Relapse** is characterized by the return to the original anxiety or insomnia symptoms for which the BZD was prescribed. It begins gradually, over weeks or months, and follows a chronic or recurrent course.
- **Rebound** implies the abrupt reappearance of the symptoms of anxiety or insomnia for which the BZD was prescribed. They typically begin within a few days to a few weeks after BZD discontinuation and last up to 3 weeks. However, these symptoms are more intense than before the treatment was initiated.
- **Withdrawal syndrome** involves new autonomic arousal symptoms that were not present before the treatment with BZD was started and that have a variable intensity and duration.

### 5.4. BZD Withdrawal Syndrome

BZD withdrawal syndrome is caused by increased sensitization of excitatory (glutamatergic) neurotransmission, which is no longer opposed by BZDs due to chronic desensitization of GABA_A_ receptors, leading to CNS hyperexcitability [36,93]. Withdrawal symptoms typically appear within 2–3 days of ceasing short-acting BZDs and 5–10 days for long-acting BZDs [15,52,94,95]. In general, withdrawal symptoms from short-acting BZDs are typically more severe than those from long-acting BZDs [95]. The course of this withdrawal syndrome is variable and can last from several weeks to several months.

- **Psychological and psychiatric symptoms** include anxiety and panic attacks, restlessness and psychomotor agitation, mood swings and depression, lack of concentration, sleeping disorders, insomnia, and nightmares.
- **Physical and sensory symptoms** include loss of appetite, tachycardia, gastrointestinal symptoms (such as nausea or vomiting, diarrhea or abdominal distension), blurred vision, dry mouth, drowsiness, muscle tension, pain, cramps and spasms, weakness, tremors, sweating, shivering, paresthesia (characterized by a feeling of “pins and needles”), tinnitus, hyperacusis, photophobia, and dysesthesia.

Regarding symptom chronology, noradrenergic symptoms have an early onset. Mood symptoms typically follow, whereas sensory and motor symptoms are more delayed, with gastrointestinal and neurological symptoms being the most delayed. Severe withdrawal symptoms include seizures, which are more frequent in polysubstance abusers and patients with a history of epilepsy, as well as with an abrupt dose reduction or cessation [95], and dopaminergic dysfunction symptoms such as delusions, visual, tactile and auditory hallucinations or illusions, depersonalization and derealization, and withdrawal delirium (see Table 4).

BZD withdrawal symptoms often occur in patients using BZDs in the long-term (more than 6 months) or at high doses (the equivalent of 100 mg of diazepam or more). In these cases, hospitalization may be required.

In addition to acute withdrawal symptoms, a protracted withdrawal syndrome has also been reported. It is associated with attenuated and prolonged symptoms such as irritability, anxiety, sleeping disorders and mood instability, which appear after several months of BZD discontinuation and remit slowly and progressively [52].

### 5.5. BZD Misuse

Withdrawal symptoms are the primary cause of BZD misuse and may contribute to subsequent abuse [94]. Table 5 summarizes the behavioral correlates of BZD misuse [15,52,93]. There are different profiles of BZD abusers and misusers [94]:

- Patients for whom BZDs were prescribed because of anxiety or insomnia, and are unable to stop the BZD once the treatment period has finished, are using BZDs for more extended periods than recommended.
- Patients for whom BZDs were indicated for any medical or psychiatric reason, but who use higher doses and for different indications than those prescribed.
- Polysubstance abusers or subjects with substance or alcohol use disorders who use BZDs to mitigate unpleasant symptoms related to drug consumption, psychiatric comorbidity or withdrawal, such as anxiety and irritability, including self-treating opioid withdrawal (“fix”) and stimulant intoxication (“come down”).
- Recreational abuse refers to individuals, normally polysubstance abusers, who use BZDs because of their direct effects, such as intoxication or euphoria. Typically, these subjects do not use BZDs alone but combined with other drugs, especially opioids, trying to potentiate the “high” or “boost”. In addition, they use BZDs over therapeutic, prefer short-acting BZDs with a rapid onset of action and use BZDs by inhalation (“snorting”) or intravenously (which is associated with other medical risks, such as infections).

### 5.6. BZD Use Disorder (BUD)

BZDs’ addictive potential is related to their ability to activate dopaminergic neurons in the ventral tegmental area by modulating GABA_A_ receptors in neighboring interneurons [15]. This phenomenon could lead to prolonged BZD use, which is closely related to the development of physiological dependence characterized by tolerance and/or withdrawal, as detailed elsewhere. It is important to emphasize that physiological dependence is one of the multiple conditions encompassing BUD according to the DSM-5-TR [10]. BUD is categorized under Sedative, Hypnotic and Anxiolytic Use Disorders and is characterized by the following conditions [52,93,94,96]:

- **Loss of control over BZD use:** thus, BZDs are used in larger doses or for a longer time than initially intended, continuing their use despite negative consequences associated with their consumption and failing during attempts to reduce or stop their use.
- **Significant time and effort spent in BZD-related activities at the expense of other rewarding activities:** for example, spending a lot of time/effort/money in acquiring the BZD or recovering from its effects, giving up on different activities in favor of BZD consumption, difficulties to focus on other activities due to craving and failure to achieve responsibilities at work, home, or academic and social settings because of BZD use.
- **Use of BZDs in risky situations:** BZDs are used recurrently in physically hazardous conditions, such as driving under the effects of these drugs or taking risks to acquire them.
- **Development of BZD physiological dependence:** development of tolerance and/or withdrawal.

Table 6 outlines diagnostic criteria for sedative, hypnotic, or anxiolytic use disorder. The diagnosis requires at least 2 of the following criteria. The disorder is considered mild if 2–3 criteria are met, moderate if 4–5 are met, and severe if 6–7 or more are met.

BZDs exhibit a unique characteristic, that is, physical and mental dependence can develop in the absence of tolerance. This phenomenon is known as low-dose or therapeutic dose dependence [15,93].

Finally, it is essential to note that several risk factors are associated with the development of a BUD. These risk factors can be categorized into drug-related, clinical practice-related, and individual risk factors as reflected in Table 7. Pharmacokinetic differences are believed to contribute to abuse and dependence potential, such that BZDs with high potency, high lipophilicity and short half-life possess the most significant abuse and dependence potential [96]. Iatrogenic dependence is more likely to occur with high doses and/or short-acting BZDs and prolonged treatment duration, which is one of the main risk factors related to BZD dependence, with a critical treatment period of 4–8 months at therapeutic doses [93,97]. In addition, abrupt discontinuation is associated with more severe withdrawal symptoms compared to gradual discontinuation. Family history or personal pre-existing or active SUD (especially alcohol, sedative-hypnotics, cannabis, opioids and stimulants) are the main individual risk factors [93,96,98].

**Figure 2:**
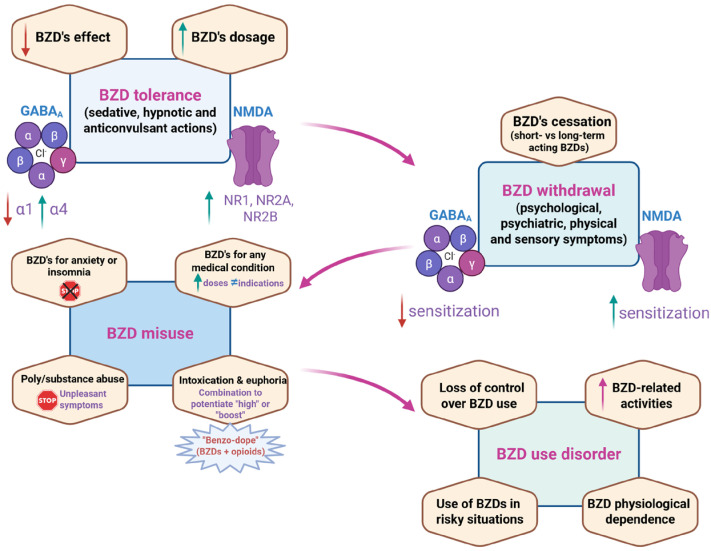
Schematic representation of the phases related to prolonged use of BZDs, including the associated main clinical and neurobiological features. GABA_A_: γ-aminobutyric acid type A receptors; NMDA: N-methyl-D-aspartate receptor; α1 and α4: alpha 1 and 4 GABA_A_ receptor subunits; NR1, NR2A and NR2B: NMDA receptor subunits.

Figure 2 summarizes the main clinical and neurobiological factors involved in the phases (i.e., tolerance, withdrawal, and misuse) that can ultimately lead to BUD.

## 6. Main Neuroadaptive Mechanisms Underlying the Adverse Effects of BZDs

The molecular mechanisms underlying the adverse effects of BZDs are not fully understood. This section aims to bring together relevant information on the involvement of the GABAergic, glutamatergic and endocannabinoid systems in the most significant adverse effects associated with long-term BZDs treatment.

### 6.1. GABAergic System

There has long been considerable interest in identifying targets of the GABAergic system, particularly those related to the GABA_A_ receptor, which may be responsible for the adverse effects of prolonged BZD use, such as sedation, tolerance, and dependence [94,99].

One of the most relevant aspects of the BZDs’ pharmacology is the development of tolerance, particularly to their sedative, hypnotic, and anticonvulsant actions, and its association with drug escalation and dependence. A great effort has been made to elucidate the mechanisms underlying this tolerance phenomenon, proposing that the GABA_A_ receptor is decoupled via several mechanisms. Modulation of GABA_A_ receptor subunit composition, alterations to the GABA_A_ receptor itself or its second messenger ligands, or variations in the receptor’s conformational state [16] are among the suggested mechanisms [100]. Interestingly, an intracellular signaling cascade involving phospholipase C isoform (PLCδ), calcium (Ca^2+^) mobilization, and activation of the Ca^2+^/calmodulin-dependent phosphatase calcineurin is critical in the BZD-induced long-term downregulation of GABAergic neurotransmission, potentially underpinning the development of pharmacological and behavioral tolerance [101].

Another key mechanism involved in BZDs tolerance is the appearance of alterations in GABA_A_ receptor subunits [102]. Chronic administration of diazepam altered the expression of individual GABA_A_ receptor subunits in different rodent brain regions [103,104,105]. Among these changes, the decrease in the α1 subunit in the hippocampus [104] or in the frontoparietal motor cortex [97], together with alterations in α5 subunit levels [104,105], could be highlighted. Furthermore, prolonged BZD exposure induces compensatory neuroadaptations in GABAA receptors, including an increased expression of the α4 subunit. This subunit, which is typically BZD-insensitive, becomes upregulated as part of a homeostatic response to sustained positive allosteric modulation, contributing to reduced drug efficacy, tolerance, and altered inhibitory signaling during chronic use and withdrawal [106].

The modification of α1 subunit expression during prolonged treatment with a BZD is one of the findings that best reflect the development of tolerance (down-regulation) from a molecular perspective and, in turn, is closely related to the onset of BZD withdrawal syndrome (up-regulation). This action could be regulated by a selective α1 GABA_A_ receptor antagonist [107]. Notably, a recent study demonstrated that diazepam-induced downregulation of the GABA_A_ receptor α1 subunit is mediated by L-type voltage-gated calcium channels (L-VGCCs). This mechanism was previously proposed [108], together with the activation of protein kinase A (PKA), and two transcription factors, the cAMP-response element-binding protein (CREB) and the inducible cAMP early repressor (ICER) [109]. This information on the intracellular signaling cascade activated by prolonged BZD exposure may help identify new molecular targets for future therapeutic interventions.

A relevant tool for elucidating the roles of different GABA_A_ subunits in the negative actions of BZDs was the development of GABA_A_ knockout and point-mutant mice [110]. Indeed, this experimental approach identified the α1 subunit as a key element in the sedative actions of diazepam [102] and demonstrated the requirement of the α5 subunit for the development of tolerance to its sedative action in mice [111]. In support of the latter finding, ligands that do not bind to the α5 subunit, such as zolpidem, have a reduced tendency to produce tolerance [112], and chronic treatment is not associated with tolerance and physical dependence [113].

Interestingly, GABA_A_ α2/α3-selective drugs do not induce tolerance, underscoring the critical role of α1 in this phenomenon and the potential to develop subtype-selective compounds [114,115]. In addition, these GABA_A_ α2/α3 selective drugs reduce the propensity to engender sedation, cognitive impairment and postural instability [116]. Therefore, the design of new, safer molecules, primarily by preventing the development of tolerance and maintaining the efficacy demonstrated by BZDs in anxiolytic, hypnotic, sedative, anticonvulsant, and muscle-relaxant effects, relies mainly on a greater understanding of the mechanisms underlying modulation of GABA_A_ receptors [117].

The rewarding properties of BZDs are closely related to their ability to increase the firing rate of dopaminergic neurons of the ventral tegmental area (VTA) through the positive modulation of α1-containing GABA_A_ receptors in nearby interneurons, which, in turn, implies a disinhibition effect that increases the dopaminergic tone in the mesolimbic system and underlies drug reinforcement [118]. Furthermore, a study with mice carrying a histidine-to-arginine point mutation in the α2 subunit, which renders it insensitive to BZDs (α2(H101R) mice), and a viral-mediated knockdown of α2 GABA_A_ receptors in the nucleus accumbens (NAc), demonstrated that the α2 subunit in the NAc is also necessary for BZD-induced rewarding properties [119].

### 6.2. Glutamatergic System

Given the molecular complexity associated with tolerance to BZDs, the pivotal role of the GABAergic system, and the delicate equilibrium with the glutamatergic system, it stands to reason that the latter may also play an instrumental role in the development of tolerance and dependence [16,120]. Indeed, some studies report alterations in excitatory glutamatergic neurotransmission targets during diazepam-induced tolerance and are associated with the appearance of signs of dependence [121]. For instance, the overexpression of the NR1 and NR2B N-methyl-D-aspartate receptor (NMDA) subunits has been proposed as a neurobiological mechanism underlying the development of diazepam tolerance [122]. Importantly, NMDA antagonists prevent the development of diazepam tolerance in rats [123,124], highlighting the crucial role of glutamatergic transmission in this phenomenon. More recently, a study analyzed the effects of prolonged alprazolam treatment on components of glutamatergic neurotransmission. An increase in NMDA receptor subunits (NR1, NR2A, NR2B), a decrease in vesicular glutamate transporter 1 (vGlut1), and differential modulation of excitatory amino acid transporters 1 and 2 (EAAT1/2) were found. These changes were associated with the onset of tolerance, along with a decrease in α1-containing GABA_A_ receptors (as previously mentioned) [125].

BZD withdrawal involves the glutamatergic system and synaptic plasticity at NMDA and α-amino-3-hydroxy-5-methyl-4-isoxazole propionate (AMPA) receptors. During withdrawal, AMPA receptors are inserted and phosphorylated at glutamatergic synapses, increasing the AMPA/NMDA transmission ratio and contributing to withdrawal symptoms [126]. To this regard, increases in AMPA and NMDA receptor expression occur upon withdrawal from diazepam [127]. Similarly, an increased incorporation of GluR1-containing AMPA receptors into the membrane, along with an increase in AMPA receptor function, was found in CA1 pyramidal neurons of rats undergoing flurazepam withdrawal. This is a common regulatory mechanism underlying synaptic remodeling and neuronal signal processing, associated with long-term potentiation (LTP) and implicated in a variety of adaptive behaviors, including drug dependence, learning, and memory [128,129].

### 6.3. Endocannabinoid System

The endocannabinoid system (ECS) has been closely involved in the modulation of emotional state, in part through interactions with the GABAergic system, which is the primary site of action of BZDs [130]. In addition, accumulating preclinical and clinical evidence suggests a dynamic interplay between the ECS and BZD-induced anxiolytic effects. In this regard, a genetic approach using male cannabinoid receptor 1 (CB1r) knockout (CB1KO) mice and wild-type (WT) littermates was carried out to determine whether deletion of CB1r alters the anxiolytic effects of bromazepam (50 or 100 microg/kg) in the light-dark box paradigm. While bromazepam markedly increased time spent in the light area in WT animals, none of the doses administered modified anxiety-like behavior in CB1KO mice. Thus, the ECS, mediated through CB1r, plays a key role in the anxiolytic effects of bromazepam [131]. In this regard, the co-administration with diazepam of cannabinoid compounds that directly activate CB1r (e.g., WIN55,212-2) or enhance the endocannabinoid tone, increasing the availability of anandamide through the inhibition of its reuptake (e.g., AM404) or enzymatic degradation by fatty-acid amide hydrolase (FAAH) (e.g., URB597), induces an additive or synergistic anxiolytic effect [132].

Our group further evaluated the role of the ECS in the main pharmacological actions and adverse effects of BZDs. First, a study in male CD1 mice using alprazolam revealed that this BZD reduced WIN-55,212-stimulated [35S]GTPɣ binding autoradiography in the amygdala and the CA1 field of the hippocampus, indicating that alprazolam significantly downregulated CB1r function in these brain areas. Furthermore, the administration of the CB1r antagonist AM251 blocked the anxiolytic effect of alprazolam and significantly reduced its sedative (ataxia, neurological severity score) and amnesic (short-term memory) actions [133]. Accordingly, more recent work further supports the idea that CB1r signaling is critically involved in the anxiolytic actions of alprazolam in male Wistar rats, which were again prevented by the CB1r antagonist AM251 in the elevated T maze (ETM) paradigm [134]. Second, a report focused on cannabinoid receptor 2 (CB2r) included male mice overexpressing this target (CB2xP), which showed reduced anxiety-like behavior across tests compared to WT mice. Interestingly, CB2xP mice displayed increased gene expression of the alpha-2 and gamma-2 subunits of the GABA_A_ receptor (GABA_A_α2 and GABA_A_γ2, respectively) in the hippocampus and amygdala, and alprazolam administration had no anxiolytic effect [135].

In summary, cannabinoid-mediated signaling through CB1r and CB2r appears to be significantly involved not only in the adverse actions of BZD but also in the anxiolytic effects, opening the door to research into possible new pharmacological strategies that modulate both the cannabinoid and GABAergic systems, to achieve a greater anxiolytic impact with a better safety profile.

Beyond their mechanistic relevance, the alterations in GABAergic, glutamatergic and endocannabinoid signaling systems described may have important implications for clinical practice in the context of BZD use and discontinuation. Changes in GABAA receptor subunit composition, particularly the downregulation of α1-containing receptors and compensatory shifts towards other subunits, may contribute to variability in tolerance, dependence and withdrawal severity between individuals. This supports the need for patient stratification based on biological factors. Concurrent upregulation of excitatory glutamatergic transmission, including NMDA receptor-mediated plasticity, provides a neurobiological rationale for gradual, individualized tapering strategies to minimize hyperexcitability and withdrawal symptoms. Furthermore, ECS dysregulation may modulate anxiety, stress responsivity and relapse vulnerability during BZD withdrawal. Together, these convergent neuroadaptive processes highlight potential therapeutic targets for adjunctive interventions and inform the development of novel pharmacological strategies designed to reduce tolerance liability and improve the safety of long-term anxiolytic and hypnotic treatments.

## 7. Preventive Strategies. Recommended Approaches to Promote an Adequate BZD Prescription, Use and Follow-Up

### 7.1. Recommended Approaches to Encourage Proper Prescription, Use and Follow-Up of BZDs

It is crucial to prevent misuse and dependence on BZDs. Several approaches have been proposed to reduce this risk [15,136]:

- BZDs should be used only for appropriate indications.
- BZDs should be employed at the minimum effective dose.
- BZDs should be prescribed in monotherapy, avoiding multiple prescriptions.
- BZDs should be only prescribed for acute disorders and for a short treatment duration. In general, it is recommended that BZD prescriptions should not exceed 2 to 4 weeks in the case of insomnia and 8 to 12 weeks in cases of anxiety, including the period for gradual tapering and withdrawal.
- When possible, non-pharmacological strategies, particularly psychological and behavioral therapies, should be considered before using BZDs in treating anxiety disorders and insomnia. Additionally, other pharmacological agents with lesser or non-abuse potential should be used as first-line treatments before considering BZD treatment.
- BZDs should be avoided in BZD-dependent high-risk patients (patients with a personal or family history of SUD, patients with chronic diseases, especially those associated with chronic pain, patients with chronic sleeping disorders and patients with psychiatric comorbidities). Additionally, BZDs should not be routinely used in special populations (in the elderly, children and adolescents, and pregnant and breastfeeding women).
- When withdrawing BZDs, abrupt discontinuation should be avoided. Gradual discontinuation is the recommended approach.
- The patient should be closely monitored for efficacy and potential adverse reactions to BZDs.
- The patient should be advised of the possibility of tolerance and dependence associated with long-term BZD use, reinforcing the importance of short-term use and BZD dose tapering. They should also be informed about side effects and withdrawal symptoms.
- The patient should be closely monitored for signs of misuse (drug-seeking behavior, need for increased dose, unwillingness to withdraw from treatment).

### 7.2. Other Pharmacological Strategies for Preventing BUD

In addition to the above recommendations, when treating anxiety disorders and chronic insomnia, other pharmacological options with lower potential for misuse and abuse should be considered:

#### 7.2.1. Anxiety Disorders

The first-line treatments for anxiety disorders include SSRIs and SNRIs. Several meta-analyses [31,137,138] have found that SSRIs and SNRIs are effective in treating anxiety disorders. Additionally, a recent meta-analysis concluded that SSRIs reduced depressive symptoms in opioid, alcohol, cocaine, cannabis, and nicotine use disorders, social anxiety symptoms in alcohol use disorder, and generalized anxiety symptoms in opioid, alcohol, cocaine, marijuana, and nicotine use disorders. SSRIs also facilitated abstinence for opioid, alcohol, cocaine, cannabis, and nicotine use; reduced craving for alcohol, cocaine, and nicotine use; and reduced alcohol use and cocaine use [139]. Other agents effective in the treatment of anxiety disorders are the anticonvulsants gabapentinoids, gabapentin and pregabalin, which target the α2δ subunits of voltage-gated calcium channels, and they are associated with a lesser potential of misuse and abuse. Both agents were found to be more effective than a placebo in reducing anxiety [140]. Recently, a meta-analysis involving 14 studies (n = 4822 patients) that evaluated the efficacy of pregabalin in the treatment of GAD demonstrated that this agent was significantly more effective than placebo in reducing anxiety in the short, medium, and long term, mainly when used in doses over 300 mg/day [141].

#### 7.2.2. Chronic Insomnia

According to European guidelines and recommendations, cognitive-behavioral therapy for insomnia (CBT-I) is considered the first-line treatment for chronic insomnia [142]. Regarding pharmacological interventions in addition to BZDs and z-drugs, orexin receptor antagonists (ORAs) and melatonin receptor agonists (MRAs) are effective for treating chronic insomnia, with no evidence of rebound insomnia or withdrawal symptoms upon discontinuation [142].

## 8. Therapeutic Strategies for BUD

### 8.1. BZD Discontinuation and Withdrawal Management

There is a consensus that BZDs should be discontinued gradually over several weeks, typically 4 to 6 weeks [15,143]. Abrupt BZD discontinuation should be avoided. There is no unique tapering strategy regarding duration or rate; individual factors, such as tolerance to withdrawal symptoms, the duration and current dose of BZD used, and the patient’s comorbidities and preferences, must be considered when planning it [143].

It has been reported that approximately 2 out of 3 patients who undergo BZD gradual tapering achieve BZD discontinuation in the short term, and 1 out of 3 successfully maintain long-term abstinence [15,143]. Factors that predict long-term abstinence include a history of prior short-term abstinence, a lower BZD dose during the initial phase of tapering, and a significant reduction in the patient’s BZD dose before beginning tapering. In contrast, a history of psychiatric comorbidity (especially anxiety and depression), comorbid alcohol use disorder, and a more severe BUD are associated with poorer outcomes [15,143].

Based on the rationale that short-acting BZDs are potentially associated with more severe dependence and withdrawal, and that withdrawal from short-acting BZDs is associated with higher dropout rates, another classical treatment strategy for BZD withdrawal has been switching from the short-acting BZD to a long-acting BZD, normally diazepam or clonazepam, at equivalent doses, followed by a gradual tapering from the long-acting BZD. However, to date, there is insufficient evidence to support the efficacy of this strategy [15].

### 8.2. BZD Inpatient Detoxification

When a patient is seeking treatment to discontinue long-term use of BZDs, the first consideration regarding withdrawal management is determining the adequate treatment setting (outpatient vs. inpatient regimen). In general, inpatient withdrawal management should be considered in the following cases [143]:

- Patients who have not been able to complete an outpatient tapering process previously.
- Patients who are taking very high BZD doses (the equivalent of >100 mg diazepam per day).
- Patients with a more severe BUD.
- Patients with a very high risk of seizures or severe withdrawal.
- Patients with another comorbid SUD, especially when there is a comorbid use of alcohol and/or opioids.
- Patients who suffer from other psychiatric or medical conditions that could be destabilized during BZD withdrawal.

Regarding BZD inpatient detoxification, in a randomized controlled study including a sample of BZD users (n = 44), no significant differences were found between a BZD tapering regimen and a symptom-triggered method of withdrawal severity, measured by the Clinical Institute Withdrawal Assessment Scale-BZDs (CIWA-B), duration of inpatient treatment, amount of BZD administered, treatment attrition, and BZD use at follow-up [144]. Oxcarbazepine, started with an initial dose of 150 mg and then increased by 150 mg/day daily during the first 4 days, 150 mg every two days up to a daily dose of 1200 mg, was used in an inpatient BZD detoxification program.

### 8.3. Adjunctive Pharmacological Agents and Biological Treatments for BZD Withdrawal and Relapse Prevention in BUD

No pharmacological agent has been specifically approved for the treatment of BZD discontinuation or relapse prevention as an adjunctive medication. However, multiple drugs have been investigated for this indication.

#### 8.3.1. Anticonvulsants

There are several potential advantages when using anticonvulsants in the treatment of BZD dependence and BZD withdrawal syndrome: (1) They reduce the likelihood of experiencing seizures, a symptom that is considered a severe complication of BZD withdrawal syndrome; (2) they block the “kindling” effect, which refers to the progressive worsening of subsequent withdrawal episodes due to neural sensitization; (3) they exhibit a low risk of abuse liability and dependence; (4) they are efficacious in treating anxiety and affective symptoms that can accompany protracted BZD withdrawal syndrome; and (5) they lack cognitive and psychomotor impairing effects.

- Valproate

A double-blind trial that assessed whether trazodone and valproate, compared to a placebo, would attenuate withdrawal symptoms and facilitate discontinuation in BZD-dependent patients (n = 78). Patients were pretreated for 1–2 weeks with trazodone (range: 100–500 mg/day), valproate (range: 500–2500 mg/day), or placebo before being tapered off their BZD at 25% per week. All treatments were continued for 5 weeks post-taper. Valproate had no significant effect on withdrawal severity or taper success at 12 weeks post-taper; however, at week 5 post-taper, 79% of patients receiving valproate remained abstinent from BZDs, which was significantly higher than in the placebo group (*p* < 0.03). Major adverse events were diarrhea, nausea, and headaches [145].

- Carbamazepine

Several studies have been conducted exploring the role of carbamazepine in the treatment of BZD withdrawal and dependence. In the first randomized, placebo-controlled trial, patients with BZD dependence (n = 40) were assigned to receive either carbamazepine (200–800 mg/day) or placebo, with a gradual BZD taper (25% reduction per week). At the end of the intervention (week 5), significantly more patients who had received carbamazepine than those who received placebo remained BZD-free. However, no statistically significant differences in withdrawal severity were observed [146]. Subsequently, a double-blind study was performed to evaluate carbamazepine for preventing BZD withdrawal in elderly patients with a comorbid anxiety disorder and BZD abuse (n = 36). The carbamazepine-treated group demonstrated a lower incidence of withdrawal symptoms (*p* < 0.01) and a more markedly reduced anxiety (*p* < 0.05) [147]. In a more recent randomized, double-blind, placebo-controlled trial, the efficacy of carbamazepine for the treatment of alprazolam withdrawal in patients with PD (n = 36) and GAD (n = 35) was explored, in which carbamazepine appeared to provide targeted support in managing alprazolam withdrawal [148].

- Pregabalin

Pregabalin (315 ± 166 mg/day) was initially explored as an adjunctive tapering treatment for improving subjective sleep quality in patients undergoing BZD withdrawal in a 12-week, prospective, open, non-controlled study conducted in patients diagnosed with BZD dependence (n = 282). Pregabalin was associated with significant and clinically relevant improvement in sleep outcomes [149]. Only one randomized, double-blinded, placebo-controlled trial has explored the efficacy of pregabalin in facilitating tapering off chronic BZD users with a lifetime diagnosis of GAD (n = 106). Patients were assigned to 12 weeks of treatment with either pregabalin (300–600 mg/day) or placebo while undergoing a gradual BZD taper at a rate of 25% per week, followed by a 6-week BZD-free phase. Results from the anxiety and withdrawal measures suggested that pregabalin substitution could be a safe and effective strategy for stopping long-term BZD therapy [150].

- Gabapentin

Only one study has evaluated the efficacy of gabapentin for the treatment of BZD dependence in outpatients in methadone maintenance treatment (n = 19). It was an 8-week, randomized, double-blind, placebo-controlled pilot trial in which gabapentin was titrated over 2 weeks to a maximum dose of 3600 mg or placebo. No significant differences were observed between gabapentin and placebo; however, the limited sample may have reduced the power to identify potential effects [151].

#### 8.3.2. Antidepressants

- Paroxetine

Two trials have been published regarding the use of the SSRI paroxetine in the treatment of BZD withdrawal. In the first randomized, double-blind, placebo-controlled trial, participants with chronic BZD use and a diagnosis of a major depressive disorder (n = 230) were assigned to receive either paroxetine (20 mg/day) or placebo during a gradual reduction in diazepam, with follow-up after 2–3 years. After 6 weeks, 75% of participants in the paroxetine group and 61% in the placebo group were successfully treated (*p* = 0.067) [152]. More recently, paroxetine (10–20 mg/day) was also evaluated and compared to placebo in outpatients with a history of BZD use for at least 3 months and no comorbid depression. The administration of paroxetine could provide benefits during BZD tapering without causing deterioration in mood among individuals without major depression [153].

- Trazodone

A small study investigated the efficacy and safety of trazodone in the treatment of BZD dependence in patients with insomnia disorder (n = 40). Patients were randomly assigned to either the trazodone or placebo groups for 3 months. Withdrawal and anxiety symptoms were significantly lower after 7 and 15 days, respectively, of treatment in the trazodone group than in the placebo group (*p* = 0.000). In addition, there was a significant improvement in sleep parameters, including total sleep time, sleep efficiency, sleep latency, and slow-wave sleep, in the trazodone group (*p* = 0.000, for all) [154].

- Tricyclic antidepressants

A first study explored the use of dothiepin for the treatment of BZD dependence. Patients (n = 88) were randomized to receive dothiepin (up to 150 mg/day) or placebo as an adjunctive treatment before BZD reduction. It was found that withdrawal symptoms were less marked in those patients allocated to dothiepin, independently of its antidepressant effect [155]. Additionally, two other trials investigated the use of imipramine for this indication. One included a sample of patients with GAD who had been long-term BZD users (n = 107) and were enrolled in a BZD discontinuation program that assessed the effectiveness of concomitant imipramine (180 mg/day) and buspirone (38 mg/day) compared to placebo in facilitating BZD discontinuation. The taper success rate in this study was significantly higher in patients who received imipramine (82.6%) than in those who received a placebo (37.5%) [156].

#### 8.3.3. Hypnotics

- Melatonin

Several studies have investigated the role of melatonin in discontinuing BZD treatment. In the first study, patients (n = 34) received either melatonin (2 mg, administered nightly for up to 6 months) or a placebo while discontinuing BZD for 6 weeks. Significantly more patients receiving melatonin discontinued BZD treatment than those receiving a placebo (*p* = 0.006). Sleep quality scores were significantly higher in the melatonin treatment group (*p* = 0.04), with a higher percentage of patients receiving melatonin maintaining good sleep quality after 6 months [157]. However, most studies have not found melatonin effective in discontinuing chronic BZD use. A double-blind, crossover control study involving patients in methadone maintenance treatment (n = 80) did not find significant differences in BZD discontinuation between melatonin (5 mg/day) and placebo. However, melatonin had an advantage in improving sleep quality, especially in patients who did not stop BZD [158]. Moreover, another placebo-controlled trial conducted in long-term BZD users (n = 38) did not demonstrate significant differences regarding discontinuation rates at the 1-year follow-up between melatonin (5 mg/day) or placebo [159].

- Zopiclone

A small, randomized, double-blind, controlled clinical trial investigated the efficacy of zopiclone in patients with insomnia and a history of hypnotic BZD (flunitrazepam) chronic use (n = 24). Both objective (polysomnographic measures and actigraphy) and subjective (self-reported sleep diaries) data consistently showed that withdrawal was considerably milder in the zopiclone group than in the flunitrazepam group [160].

#### 8.3.4. Anxiolytics

- Alpidem

Alpidem, an anxiolytic drug from the imidazopyridine family, has also been investigated in two small trials as an adjunctive treatment for BZD discontinuation with conflicting results. In the first study, chronic BZD users (n = 25) were gradually tapered from the BZD used and substituted for alpidem (25 mg twice daily) or placebo. Anxiety increased in the alpidem but not in the placebo patients [161]. However, a 6-week, multicenter, double-blind, randomized, placebo-controlled trial was carried out in outpatients suffering from generalized anxiety or adjustment disorder with an anxious mood (n = 173). Patients abruptly discontinued BZD use and were treated with alpidem (50 mg twice or three times daily) or a placebo. Alpidem showed therapeutic potential in preventing BZD withdrawal syndrome [162].

- Buspirone

Buspirone is a serotonin 5-HT1A receptor partial agonist that exerts anxiolytic effects, and it is primarily used to treat anxiety disorders, particularly GAD. Four small trials have investigated the efficacy of the anxiolytic buspirone in treating BZD dependence, with contradictory results. A multicenter, double-blind, placebo-controlled study was conducted to evaluate the safety of buspirone in combination with alprazolam during the gradual tapering of alprazolam in BZD-dependent patients (n = 72). It was found that there was significantly greater anxiety in the placebo group and reduced manifestations of abstinence in the buspirone group [163]. Additionally, in a later study in which long-term BZD users (n = 24) were allocated randomly to treatment with either buspirone (15 mg/day) or a placebo, before tapering off the BZD, buspirone-treated patients tended to have lower anxiety levels than placebo-treated patients [164]. However, in another study that included outpatients on long-term BZD treatment (n = 24) who were randomly allocated to either a placebo or buspirone undergoing withdrawal over 10 weeks, buspirone exhibited some anxiolytic effect, although insufficient to assist BZD withdrawal [165].

- Propranolol

Propranolol, a β-blocker with anxiolytic properties, has been explored in one randomized, double-blind, placebo-controlled study including chronic lorazepam or diazepam users (n = 40). Propranolol (60–120 mg/day) did not affect the dropout rate or the incidence of withdrawal symptoms but significantly reduced their severity in patients completing the study [166,167].

- Captodiame

Captodiame is a first-generation antihistamine that is used as a sedative and anxiolytic agent. Its efficacy in preventing the emergence of BZD withdrawal symptoms has been explored in a 6-week, randomized, double-blind, placebo-controlled trial in individuals presenting mild to moderate anxiety and treated for at least 6 months with a stable dose of BZD (n = 81) who received either captodiamine (150 mg/day) or placebo while gradually tapering BZD [167,168].

#### 8.3.5. Other Agents

- Flumazenil

Flumazenil is a GABA_A_ receptor antagonist used for rapid BZD detoxification. It appears to normalize BZD receptors, returning them to a baseline state, upregulate GABA_A_ receptors, exert a weak agonist action, and suppress BZD withdrawal symptoms. Several studies have investigated flumazenil’s efficacy and safety in treating BZD withdrawal. A small initial first trial included chronic BZD (flunitrazepam or lormetazepam) abusers (n = 36). Patients were randomly assigned to receive either flumazenil or a placebo during abrupt discontinuation of BZDs. It was found that, compared to patients treated with flumazenil, those receiving a placebo showed a significant increase in mean withdrawal scores (*p* < 0.001). Additionally, no severe withdrawal symptoms, such as seizures, were observed after the flumazenil infusion [169].

The same group conducted a further experiment in which flumazenil (1 mg intravenously twice daily for 8 days) was compared with oxazepam tapering (from 120 mg) and placebo in the control of BZD withdrawal symptoms in BZD-dependent patients (n = 50). Flumazenil was shown to immediately reverse BZD effects on balance tasks and significantly reduce withdrawal symptoms on both self-reported and observer-rated withdrawal scales and craving compared to oxazepam and placebo [170].

- Lithium

Lithium gluconate has been investigated in a French multicenter, randomized, double-blind, placebo-controlled study. Outpatients receiving BZD for at least 6 months (n = 244) were randomly allocated to lithium or a placebo during a gradual tapering of their BZD. Both lithium and placebo were associated with high BZD discontinuation rates (over 60%) without significant differences between them. Additionally, no significant differences between lithium and placebo were observed regarding anxiety and withdrawal symptoms [167,171].

- Progesterone

Progesterone has been explored for the treatment of BZD withdrawal symptoms and for facilitating BZD discontinuation in a single double-blind, placebo-controlled study, based on the suggested barbiturate-like modulation of GABAergic function by some of its metabolites. BZD-dependent patients with at least 1 year of continuous daily use (n = 40) were assigned to treatment with either placebo or progesterone (titrated to a mean daily dose of 1983 mg for 3 weeks), while BZD was tapered by 25% per week. There were no significant differences between progesterone and placebo regarding the severity of taper withdrawal, control of anxiety symptoms and BZD drug-free after 12 weeks [172].

- Magnesium aspartate

To evaluate the efficacy of alpha-beta L-Aspartate Magnesium in discontinuing long-term BZD use, a 3-month, randomized, double-blind, placebo-controlled trial was conducted among chronic BZD users (n = 144). No significant differences were found regarding BZD discontinuation, BZD cessation without withdrawal or anxiety symptoms or BZD relapse [173].

- Cyamemazine

Cyamemazine is a first-generation antipsychotic drug of the phenothiazine class with anxiolytic properties. Its efficacy in facilitating BZD withdrawal was investigated in a trial involving patients who had been treated with BZDs for at least 3 months (n = 168). No statistically significant differences between treatment groups were found regarding rebound anxiety, although there was an advantage in favor of cyamemazine for relapsing to BZD use [174].

- Ondansetron

Ondansetron, an antagonist of the 5-HT3 receptor, was evaluated in a randomized, double-blind trial as an adjunctive medication to help discontinue BZDs in long-term users (n = 108). Patients received either ondansetron (2 mg twice daily) or a placebo while tapering their BZD over 6 weeks. The percentage reduction in BZD daily dosage at all time points in the treatment trial was similar for the ondansetron and placebo groups, and ondansetron had no significant effect on the severity of withdrawal symptoms or levels of anxiety [175].

To summarize, valproate and TCA may have a potentially positive effect on BZD discontinuation. On the other hand, withdrawal symptoms could be potentially attenuated by pregabalin, captodiame, paroxetine, tricyclic antidepressants, and flumazenil. Regarding the reduction in anxiety symptoms, carbamazepine, pregabalin, captodiame, paroxetine, and flumazenil are the most effective. In addition, valproate and cyamemazine could be associated with a reduction in the proportion of patients relapsing in BZD use. However, clinical experience with the drugs described in this section is limited to varying degrees, and their efficacy is somewhat controversial. It is therefore essential to tailor pharmacological treatment to each patient’s symptoms and characteristics. Furthermore, it is crucial to continue exploring new pharmacological strategies that could enhance the efficacy and safety of therapy for BUD.

#### 8.3.6. Repetitive Transcranial Magnetic Stimulation

Repetitive transcranial magnetic stimulation (rTMS) is a non-invasive neuromodulation technique that uses magnetic pulses to stimulate specific areas of the brain, with the potential to alleviate depressive and anxious symptoms and improve sleep quality in BZD-dependent individuals. A retrospective study including psychiatric patients treated with high-frequency rTMS over the left dorsolateral prefrontal cortex showed that this technique effectively reduced symptoms of depression and anxiety, particularly in patients taking BZDs [176]. A recent 12-week, randomized, controlled trial investigated the efficacy of rTMS in BZD-dependent inpatients (n = 40). Although significant improvements (*p* < 0.05) were observed in both groups regarding anxiety symptoms, depressive symptoms and sleep quality, rTMS added to BZD discontinuation conventional treatment exhibited superior improvements in all these measures, especially in weeks 8 and 12 of follow-up [177].

### 8.4. Psychological Treatments in BUD

Several psychological therapies have been proposed for the treatment of BUD. The aims of these interventions are facilitating BZD withdrawal, facilitating further abstinence (relapse prevention), and treating the underlying disorders for which the BZD were prescribed [15,178,179]:

- **Psychoeducation and minimal brief interventions.** Minimal, brief interventions in primary care can facilitate an initial reduction in BZD use.
- **Motivational interviewing.** It is based on the transtheoretical model of Prochaska and DiClemente.
- **Cognitive-behavioral therapy (CBT).** It is the most effective psychological treatment for BZD dependence. Its main objective is to prevent BZD relapse.
- **Mindfulness-based interventions (MBI).** In mindfulness-based relapse-prevention interventions, participants develop skills to manage discomfort and negative emotions, thereby broadening their repertoire of adaptive responses to situations that previously led to BZD use.
- **Other psychological treatments.** Other psychological therapies that have been explored in the management of BUD include self-control training, exposure to BZD cues to induce and manage craving, marital and familiar therapy (which considers that the patient suffering from a BUD reflects a dysfunctional family system such that the addictive behavior would be an attempt to regulate or control these dysfunctional relationships), and psychodynamic or psychoanalytic therapies (which consider addition as a failed attempt to self-healing and focus on frustration, poor problem-solving skills or low tolerance to negative emotions that could increase anxiety and hinder abstinence).
- **Non-pharmacological strategies for treating sleeping disorders.** They are based on stimulus control and sleep hygiene education, in which the patient learns to maintain a regular wake-sleep pattern, engage in relaxing activities before bedtime, and avoid stimulating activities.

Multiple studies and meta-analyses have demonstrated that the effectiveness of gradual deprescription of BZDs increases when non-pharmacological therapies are added, both in the short- and long-term [180,181,182]. Specifically, CBT is the psychological therapy that has the most evidence in the treatment of BUD. CBT was first shown to be effective when added to BZD gradual tapering in reducing BZD use in the short term for long-term BZD users with coexisting anxiety disorders, chronic insomnia or BZD dependence [180]. Regarding the efficacy of CBT in discontinuing BZDs among patients with anxiety disorders, a recent meta-analysis found that the proportion of patients discontinuing BZDs was significantly higher in the CBT plus BZD gradual tapering group than in the BZD gradual tapering alone group, both in the short term (3 months) and long term (6 to 12 months after allocation) [181]. Table 8 and Table 9 summarize treatment strategies in BUD.

## 9. Concluding Remarks

BZDs maintain their standing as globally prevalent psychotropic substances, valued for their anxiolytic, hypnotic, anticonvulsant, and myorelaxant properties, fundamentally achieved through positive allosteric modulation of the ionotropic GABA_A_ receptor. Despite this recognized clinical utility, the widespread and often prolonged use of these agents—typically exceeding 8 to 12 weeks—constitutes a significant public health imperative due to the inherent risk of severe adverse outcomes, including cognitive impairment, motor incoordination, tolerance, and physical dependence, ultimately leading to BUD.

The transition to dependence is mediated by complex neurobiological substrates, notably the downregulation of GABA_A_ receptor subunits (such as α1) and a concurrent compensatory sensitization of excitatory glutamatergic systems, with the pivotal role of the ECS. This neuroadaptation mechanism is critical to understanding the development of tolerance. Furthermore, the rewarding properties that contribute to the addictive potential of BZDs are linked to the positive modulation of α1-containing GABA_A_ receptors in VTA interneurons, thereby increasing dopaminergic tone in the mesolimbic system.

Given these risks, preventive strategies are paramount, emphasizing short-term prescriptions and minimum effective dosing, and prioritizing first-line pharmacological agents with lower abuse potential, such as SSRIs and SNRIs, for anxiety and related disorders. Where dependence is established, therapeutic management is heterogeneous and complex. The consensus approach involves a gradual dose taper, often preceded by pharmacological substitution with a long-acting BZD such as diazepam or clonazepam. CBT represents the most effective psychological intervention when added to gradual tapering, proving crucial for facilitating BZD discontinuation and relapse prevention. Adjunctive pharmacological agents, including certain anticonvulsants (e.g., pregabalin, valproate) and tricyclic antidepressants, show potential in attenuating withdrawal symptoms and anxiety, highlighting the need for further exploration into targeted pharmacotherapies.

Future efforts must continue to dissect the molecular mechanisms underlying BZD toxicity to facilitate the design of safer, subtype-selective molecules, particularly those targeting α2 and/or α3 subunits while avoiding the α1-mediated sedative and tolerance effects.

## Figures and Tables

**Table 1:** Abundance and localization of common GABA_A_ receptor subtypes in the brain [16].

Subtype	Frequency	Localization
***α*_1_ *β*_2_ *γ*_2_**	Major (60%) synaptic	Cerebral cortex (layer I–VI), hippocampus, striatum, cerebellum, amygdala, brainstem
***α*_2_ *β_n_γ*_2_**	Minor (15–20%) synaptic	Cerebral cortex (layers I–IV), hippocampus, striatum, hypothalamus, amygdala
***α*_3_ *β_n_γ*_2_**	Minor (10–15%) synaptic	Cerebral cortex (layers V–VI), hippocampus, cerebellum, amygdala, brainstem (including raphe nuclei and locus coeruleus), spinal cord
***α*_4_*β_n_δ*/*γ***	Minor (<10%) extrasynaptic	Hippocampus (dentate gyrus), thalamus, cortex
***α*_5_ *β_n_γ*_2_**	Minor (<5%) extrasynaptic	Cerebral cortex, hippocampus, amygdala, hypothalamus, spinal cord
***α*_6_*β_n_γ*_2_ /*δ***	Minor (<5%) extrasynaptic	Cerebellum

**Table 2:** Classification of BZDs according to the elimination half-life, including main pharmacokinetic characteristics and clinical therapeutic applications. ADMON: administration; BZD: benzodiazepine; i.v.: intravenous; p.o.: *per* os; PP: plasmatic proteins; VD: volume of distribution; -: no available data [20,28].

BZD	Route of ADMON	Onset of Action	Bioavailability (%)	VD (L/Kg)	PP Binding (%)	Elimination Half-Life (h)	Active Metabolites	Clinical Therapeutic Use
**Ultra-Short Acting**								
**Midazolam**	p.o.	3–5 min	30–70	50.2	96–98	1.5–2.5	no	Anticonvulsant, sedative
**Triazolam**	p.o.	15–30 min	90–100	0.57–0.86	80–94	1.5–5.5	no	Hypnotic
**Short Acting**								
**Alprazolam**	p.o.	1 h	80	0.72	80	12–15	no	Anxiolytic
**Lorazepam**	p.o.	20–40 min	90	1–1.3	85	10–20	no	Anxiolytic, hypnotic
**Lormetazepam**	p.o.	20 min–1 h	80	2.8–4.6	85	12–20	no	Hypnotic
**Oxazepam**	p.o.	20 min–1 h	-	-	97	6–10	no	Anxiolytic
**Brotizolam**	p.o.	30 min–1 h	-	-	-	3–6	yes	Hypnotic
**Remimazolam**	i.v.	Very fast	-	-	-	-	yes	Anesthetic inducer, conscious sedation
**Intermediate Acting**								
**Bromazepam**	p.o.	2–3 h	60	50	70	11–22	no	Anxiolytic
**Ketazolam**	p.o.	2.5–3 h	90–100	193.7	93	15–52	yes	Anxiolytic, muscle relaxant
**Flunitrazepam**	p.o.	0.5–2 h	-	-	-	-	yes	Hypnotic
**Loprazolam**	p.o.	0.5–4 h	90	-	-	-	no	Hypnotic
**Long Acting**								
**Clobazam**	p.o.	30–120 min	100	100	80–90	36–42	yes	Anxiolytic, Anticonvulsant
**Clonazepam**	p.o.	20–60 min	90	3	82–86	18–50	no	Anxiolytic, Anticonvulsant
**Clorazepate**	p.o.	30–60 min	50	0.93–1.27	98	36–60	yes	Anxiolytic, hypnotic
**Clordiazepoxide**	p.o.	15–45 min	100	0.27–0.33	85–95	40–100	yes	Hypnotic
**Diazepam**	p.o.	10–45 min	80–100	0.8–1	98	40–200	yes	Anxiolytic, anticonvulsant, muscle relaxant, sedative
**Flurazepam**	p.o.	20 min	30	1.4	97	50–160	yes	Hypnotic
**Quazepam**	p.o.	60 min	-	-	-	40	yes	Hypnotic
**Medazepam**	p.o.	1–2 h	-	0.9–1.2	98–99	-	yes	Anxiolytic

**Table 3:** BZD discontinuation syndromes. For each syndrome, the characteristics of the symptoms, the comparative severity to the original symptoms for which the BZD was prescribed, and the progression over time are shown.

Syndrome	Symptoms	Severity of Symptoms Compared to the Original Symptomatology	Progress
**Relapse**	Similar to originals	The same	Gradual onset No decline over time
**Rebound**	Similar to originals	Higher intensity	Abrupt onset Temporal course
**Withdrawal**	New symptoms	Variable	Variable 2–4 weeks duration

**Table 4:** Symptoms associated with BZD withdrawal syndrome.

Persistent Symptoms	Frequent Symptoms	Rare Symptoms
**Anxiety** **Insomnia** **Restlessness** **Agitation** **Irritability** **Muscle tension**	Nausea Rhinitis Diaphoresis Lethargy Hyperacusis Blurred vision Depression Hyperreflexia Ataxia	Psychosis Seizures Tinnitus Confusion Delusions Hallucinations

**Table 5:** A detailed overview of the most common and frequent clinical behavioral features associated with BZD misuse (modified from Soyka, 2017 [15]; Guina & Merrill, 2018 [93]).

Individuals Who Misuse BZDs Exhibit the Following Features:
▪ Taking BZDs, even prescribed at therapeutic and low doses, for months or years. ▪ Progressively need BZDs to carry out daily activities. ▪ Continuing use of BZDs even when the original indication for which the BZD was prescribed has disappeared. ▪ Difficulties with cutting down or stopping BZDs due to withdrawal symptoms. ▪ Having anxiety between doses or experiencing a craving for the next dose when taking short-acting BZDs. ▪ Contacting their doctor frequently to obtain regular prescriptions. ▪ Becoming anxious if the next prescription is not rapidly available. ▪ Carrying their tablets and taking extra doses when they anticipate stressful events. ▪ Increasing the doses from the original prescription. ▪ Experiencing anxiety symptoms, panic attacks, agoraphobia, insomnia, depression or physical symptoms despite continuing to take BZDs. ▪ Obtaining BZDs from several providers without informing them of the others (“doctor-shopping”), frequent visits to the emergency department and “losing prescriptions.” ▪ Using private prescriptions. ▪ Giving, selling or trading BZDs to others. ▪ Taking hypnotic BZDs during the day.

**Table 6:** Diagnostic and Statistical Manual of Mental Disorders, 5th edition (text revision), diagnostic criteria for sedative, hypnotic, or anxiolytic use disorder [10].

A Problematic Pattern of Sedative, Hypnotic, or Anxiolytic Use Leading to Clinically Significant Impairment or Distress, as Manifested by at Least Two of the Following, Occurring Within 12 Months:
Taking the drug in higher amounts or over a longer period than was intended. There is a persistent desire or unsuccessful efforts to reduce or control use. A great deal of time is spent on activities necessary to obtain or use the drug or recover from its effects. Craving, or a strong desire or urge to use the drug. Recurrent use of the drug results in failing to fulfill significant obligations at work, school, or home. Continued use of the drug despite having persistent or recurrent social or interpersonal problems caused or exacerbated by its effects. Important social, occupational, or recreational activities are given up or reduced because of the use of the drug. Recurrent use in physically hazardous situations. Drug use continues despite the awareness of having persistent or recurrent physical or psychological problems that are likely to have been caused or exacerbated by the drug. Tolerance, as defined by (a) a need for markedly increased amounts of the drug to achieve intoxication or the desired effect, and (b) a markedly diminished effect with continued use of the same amount of the drug. Withdrawal, as manifested by either (a) the characteristic withdrawal syndrome for sedatives, hypnotics, or anxiolytics, or (b) sedatives, hypnotics, or anxiolytics taken to relieve or avoid withdrawal symptoms.

**Table 7:** Risk factors associated with the clinical development of BZD dependence [93,96,97].

Category	Risk Factors
**Drug-Related**	▪ High-potency BZD ▪ Ultra-short or short-acting BZD ▪ Lipophilic BZD with faster absorption
**Clinical Practice-Related**	▪ Using high dosages ▪ Prolonged treatment duration ▪ Early onset of use ▪ Combined or consecutive administration of BZD with other drugs ▪ Reduction schedule (abrupt discontinuation)
**Individual**	▪ Personal or family history of Alcohol Use Disorder or another SUD ▪ History of chronic medical conditions, particularly pain disorders ▪ History of psychiatric disorders ▪ Chronic sleeping disorders (insomnia)

**Table 8:** Adjunctive pharmacological treatments for different clinical stages associated with BUD, including specific recommendations regarding safety and worsening (modified from Baandrup et al., 2018 [167]).

Intervention	Pharmacological Agents Suggested
**BZD discontinuation**	Valproate Tricyclic antidepressants
**BZD withdrawal**	Pregabalin Captodiame Paroxetine Tricyclic antidepressants Flumazenil
**Anxiety**	Carbamazepine Pregabalin Captodiame Paroxetine Flumazenil
**Proportion of relapse to BZD use**	Valproate Cyamemazine
**Safety issues**	Flumazenil: It can precipitate severe withdrawal syndrome
**Worsening outcomes**	Alpidem: It worsens discontinuing BZD and the intensity of withdrawal symptoms. Magnesium aspartate: It decreases the rates of BZD discontinuation.

**Table 9:** Therapeutic strategies for managing BZD withdrawal (modified from Soyka, 2017 [15]).

Situation	Treatment Strategy	Level of Evidence
**BZD discontinuation**	Gradual tapering over several weeks or months	High
**Psychotherapy**	Cognitive-behavioral therapy	Good
**Use of >1 BZD**	Switch to only one BZD (diazepam) for detoxification.	Good
**Concomitant pharmacotherapy for BZD discontinuation**	Valproate Tricyclic antidepressants	Moderate/Low
**Concomitant pharmacotherapy for BZD withdrawal**	Pregabalin Captodiame Paroxetine Tricyclic antidepressants Flumazenil	Moderate/Low
**Choice of BZD for** **detoxification**	Switch to a long-acting BZD	Low

## Data Availability

No new data were created or analyzed in this study. Data sharing does not apply to this article.
